# Extrusion-based additive manufacturing of fungal-based composite materials using the tinder fungus *Fomes fomentarius*

**DOI:** 10.1186/s40694-021-00129-0

**Published:** 2021-12-21

**Authors:** Huaiyou Chen, Amanmyrat Abdullayev, Maged F. Bekheet, Bertram Schmidt, Isabel Regler, Carsten Pohl, Cekdar Vakifahmetoglu, Mathias Czasny, Paul H. Kamm, Vera Meyer, Aleksander Gurlo, Ulla Simon

**Affiliations:** 1 Chair of Advanced Ceramic Materials, Institute of Material Science and Technology, Faculty III Process Sciences, Technische Universität Berlin, Straße des 17. Juni 135, 10623 Berlin, Germany; 2 Chair of Applied and Molecular Microbiology, Institute of Biotechnology, Faculty III Process Sciences, Technische Universität Berlin, Straße des 17. Juni 135, 10623 Berlin, Germany; 3 Department of Materials Science and Engineering, Izmir Institute of Technology, 35430 Urla, Izmir, Turkey; 4 Institute of Applied Materials, Helmholtz-Zentrum Berlin für Materialien und Energie, Hahn-Meitner-Platz 1, 14109 Berlin, Germany

**Keywords:** Fungi, Mycelium, Additive manufacturing, Extrusion, Alginate, Freeze-drying, Micro-computed tomography, Tinder fungus

## Abstract

**Background:**

Recent efforts in fungal biotechnology aim to develop new concepts and technologies that convert renewable plant biomass into innovative biomaterials. Hereby, plant substrates become metabolized by filamentous fungi to transform them into new fungal-based materials. Current research is thus focused on both understanding and optimizing the biology and genetics underlying filamentous fungal growth and on the development of new technologies to produce customized fungal-based materials.

**Results:**

This manuscript reports the production of stable pastes, composed of *Fomes fomentarius* mycelium, alginate and water with 71 wt.% mycelium in the solid content, for additive manufacturing of fungal-based composite materials. After printing complex shapes, such as hollow stars with up to 39 mm in height, a combination of freeze-drying and calcium-crosslinking processes allowed the printed shapes to remain stable even in the presence of water. The printed objects show low bulk densities of 0.12 ± 0.01 g/cm^3^ with interconnected macropores.

**Conclusions:**

This work reports for the first time the application of mycelium obtained from the tinder fungus *F. fomentarius* for an extrusion-based additive manufacturing approach to fabricate customized light-weight 3D objects. The process holds great promise for developing light-weight, stable, and porous fungal-based materials that could replace expanded polystyrene produced from fossil resources.

**Supplementary Information:**

The online version contains supplementary material available at 10.1186/s40694-021-00129-0.

## Background

Environmental pollution and the depletion of petroleum resources necessitate the development of new renewable and eco-friendly materials to achieve a sustainable future. Fungal mycelium is exceptionally promising because it can be produced on the basis of a wide variety of organic substrates, such as agricultural residues, it is fully biodegradable and has low density, low production cost, and low processing energy input [1, 2]. This makes fungal mycelium a sustainable option for the production of various materials, including materials for packaging [3], construction [4], sound absorption [5–7], flame-retardation [8], medication [9], filtration [10], and as a substitute for leather [11, 12]. The ability to degrade lignocellulosic plant biomass by the fungal division Basidiomycota including white- and brown-rot fungi, combined with their flexible but strong cell walls make them ideal candidates for the development of new cell factories for the production of a wide variety of materials [13]. Hereby, both pure fungal mycelium and composites consisting of fungal mycelium and plant biomass are of interest. Pure fungal mycelium can be obtained by cultivating fungi in liquid or on solid organic substrates [14, 15]. The exterior surface of fungi comprises glucans that function as mucilage as well as water-repellent hydrophobins, while the inner layer is rich in chitin microfibrils, which provide stiffness and is covalently cross-linked with other polysaccharides like glucans, which provide elasticity to the fungal cell wall [13, 16, 17].

*Fomes fomentarius*, the tinder fungus, is a white-rot fungus that is widespread in the northern hemisphere of the earth and equally domestic to Europe, Asia, and North America. It is famous for being a medicinal fungus in Traditional Chinese Medicine and Traditional European Medicine and its fruiting bodies were harvested for the production of textiles and wound pads in Germany till the eighteenth century. Hyphae of *F. fomentarius* grow well under laboratory conditions on different by-products from agriculture and forestry including hemp, raps straw and aspen sawdust, and can be used to produce fungal-based composite materials as most recently reported [18]. Hereby, *F. fomentarius* becomes cultivated on solid lignocellulosic substrates (such as straw or wood chips) that have been packed into a mold shape. In contrast to molding-based techniques, additive manufacturing (AM), known as 3D printing, does not rely on a specific mold, and therefore allows the fabrication of complex parts with unusual geometry. This is highly advantageous when object design and geometry frequently change, as in the case for furniture, niche product packaging, and architecture. For instance, Bhardwaj et al. [19] reported AM of a two-step colonized biomass-waste-fungal composite material that could be considered for packaging and building applications. Blast Studio has been working on a similar approach using take-away waste biomass and fungi for manufacturing unique art and furniture pieces in limited editions with AM [20]. Goidea et al. [21] built composite materials for architecture from living fungi, plant biomass, and clay and Krayer et al. [6] mixed mycelium with a vegetative substrate and printed it into the desired form to be used as a sound absorber. NASA's Ames Research Center’s myco-architecture project or the Growing Fungal Structures in Space project of ESA considers fungal-based materials in combination with AM even for Moon and Mars exploration for future habitats [22, 23]. Although AM of fungal-based materials shows a huge potential for many applications from an environmental perspective, very limited research has been conducted into this topic so far. Furthermore, the addition of solid substrates, like sawdust, which is typically used for AM in fungal-based materials, and the fibrous mycelium itself, makes the adaptation of AM challenging, especially in printing high-precision parts [24].

In the present work, the focus was on the development of an extrusion-based AM method for shaping mycelium-based 3D printed objects by using inactive pure mycelium from *F. fomentarius*. We aimed at using its pure fungal biomass and using renewable biopolymer alginate, which can be obtained from seaweed or bacteria, to adjust the viscosity of the mycelium slurry to make the mycelium extrudable through fine nozzle diameters so that it could be printed. Our rationale was that by introducing the highly viscous alginate biopolymers, the dispersion medium could in turn potentially exert sufficient force to drag the mycelium out of the nozzle, i.e. the viscosity of the mycelium slurry can be tuned in such a way, that the paste can be extruded. Specifically, our goals were (i) the development of a mycelium-biopolymer paste for extrusion-based AM, (ii) AM fabrication of 3D objects by using the newly developed paste, and (iii) the investigation of process parameters, such as nozzle diameter, drying, and cross-linking procedures on the quality of the printed 3D objects.

## Results and discussion

### Paste development with highly viscous alginate and fungi

**Fig. 1:**
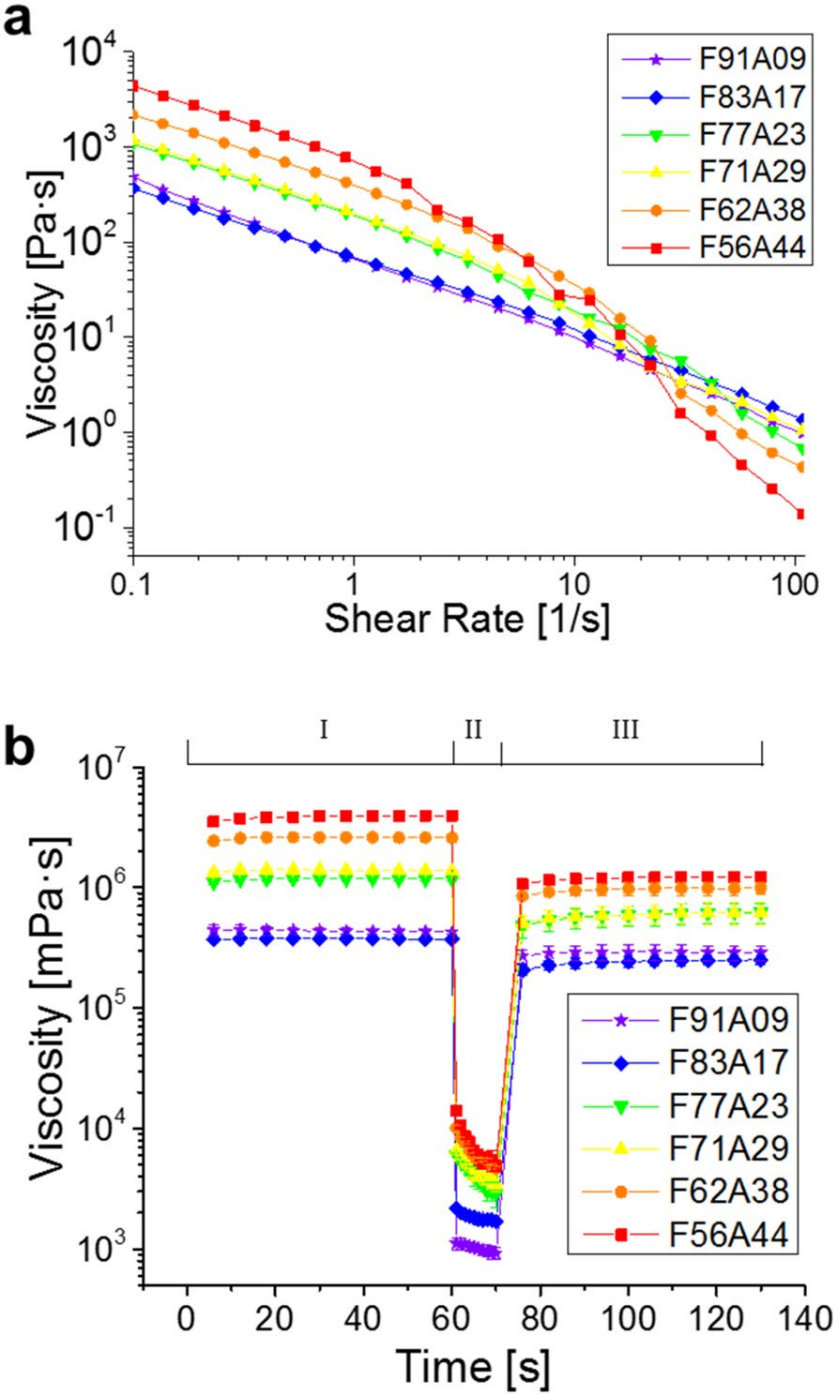
Rheological properties of mycelium-alginate pastes: **a** results of the steady-state flow tests and **b** results of the three-step flow tests (sample abbreviations see "Methods")

Criteria for obtaining the optimal paste formula for printing include the rheological properties of the paste, filament homogeneity, and stackability, as well as the results of the collapse tests. For pure alginate pastes, an increase in viscosity was observed with increasing alginate content. All prepared pastes showed shear-thinning characteristics, i.e., paste viscosity decreased with increasing shear rate, which was more pronounced for the higher alginate contents (Additional file 1: Fig. S1) [25]. Due to the addition of mycelium, the viscosity of mycelium-alginate pastes increased (Fig. 1a). All mycelium-alginate pastes demonstrated shear-thinning behavior. Although the pastes with higher mycelium content possess higher steady-state viscosity, the shear-thinning behavior was more pronounced at higher alginate contents. In accordance with the pure alginate pastes, the viscosity was decreased for lower alginate contents within the pastes.

The three-step flow tests (Fig. 1b) showed the viscosity recovery values for all pastes by comparing the viscosity value at a shear rate of 0.1 s^−1^ at step III to the initial value at step I and thus before and after being subjected to a high shear rate of 100 s^−1^. The percentage of viscosity recovery decreased as alginate content was increased relative to the mycelium. This indicates that the higher contents of viscous alginate do not necessarily lead to better printing quality (for samples F91A09, F83A17, F77A23, F71A29, F62A38, and F56A44, the viscosity recovery rates were found to be 63%, 55%, 41%, 37%, 33%, and 27%, respectively), although they can increase initial viscosity (Fig. 1b) and thus make mycelium pastes printable. One hypothesis could be that the fungal hyphae were initially entangled with each other, which, together with the high concentration of alginate, resulted in a high starting viscosity. After a high shear force was applied, the entanglement was probably opened. Thus, the higher the viscosity of alginate, the more fungal hyphae were untangled. Eventually, the viscosity did not return to its original state because there were no entangled hyphae left.

**Fig. 2:**
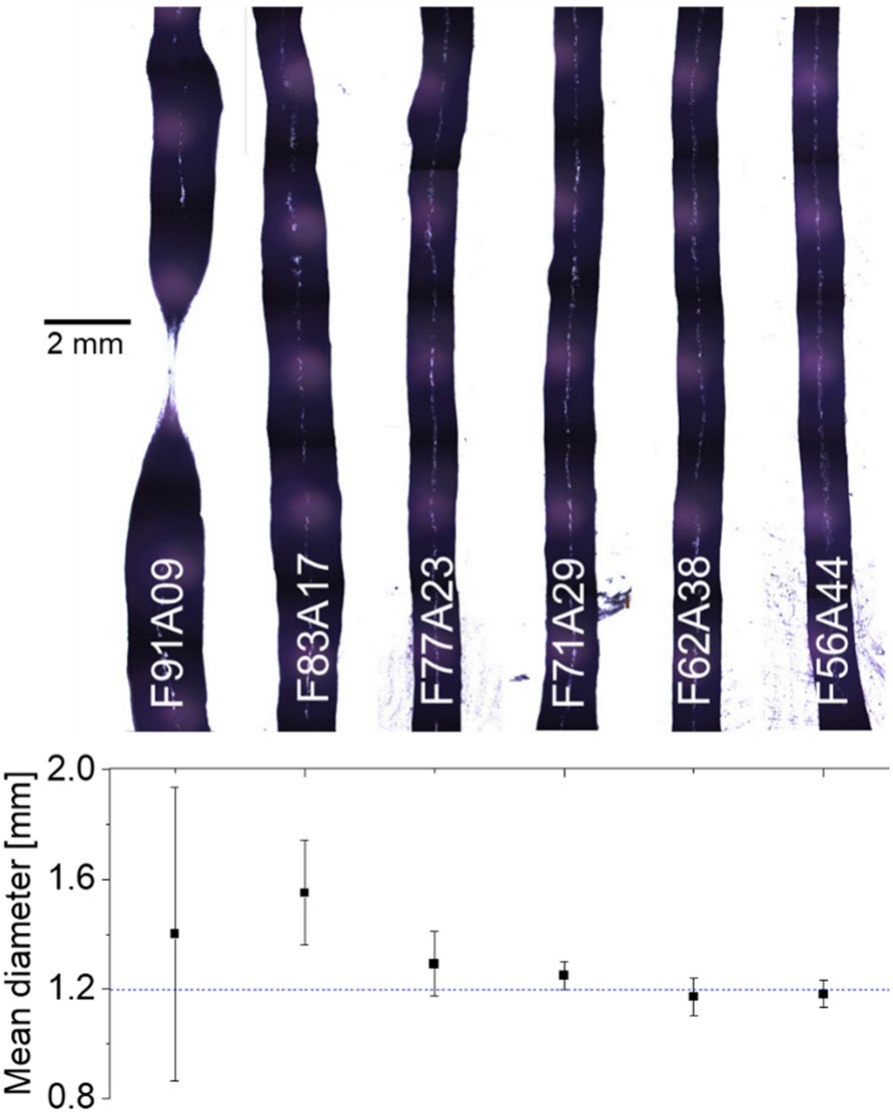
Microscopic images of filaments extruded with 1.2 mm nozzle along with their mean diameters. The images were obtained from multiple shots and then stacked together to eliminate overlap

During filament homogeneity testing, it was noticed that printed filaments with the pastes with alginate contents lower than 29 wt.% were less confined by the diameter of the nozzle than other pastes and showed less homogeneous width distributions (Fig. 2).

**Fig. 3:**
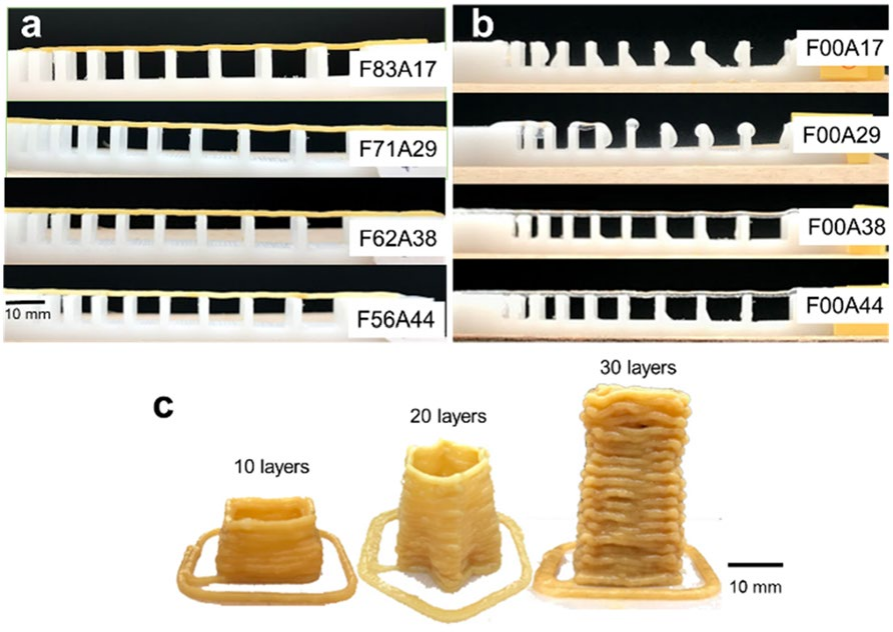
**a**, **b** Filament collapse tests (nozzle diameter 1.2 mm, layer height 1.0 mm): optical images of filaments extruded from **a** mycelium-alginate composite and **b** pure alginate gel. **c** Stackability tests: optical images of objects (see Fig. 9b) printed with the F71A29 paste with 10, 20 and 30 layers (nozzle diameter 1.6 mm, layer height 1.3 mm, printing speed 10 mm/s)

All mycelium-alginate composite samples maintained the structural integrity of their filaments when subjected to the maximum gap distance of 10 mm in the filament collapse test (Fig. 3a). None of the studied filaments collapsed between the pillars, suggesting that they provided good support when printing hollow structures. Compared with the results of low concentrated pure alginate (Fig. 3b), for which an insufficient strength of the pastes resulted in structural collapse, the mycelium fibers in the composite pastes sufficiently reinforced the filament. The height of a printed object reflects directly the stackability of the pastes. Stackability tests showed that pastes could stack up well to 39 mm when their alginate content was greater than 29 wt.%, whereas F83A17 filament collapses when piled to 20 layers.

Summarizing, the pastes with F71A29 formulation showed good rheological properties and led to comparatively homogeneous extruded filaments with high stackability without collapsing; this formulation also had a relatively high mycelium content and was thus chosen for further extrusion-based AM study.

### AM of mycelium-alginate composites

**Fig. 4:**
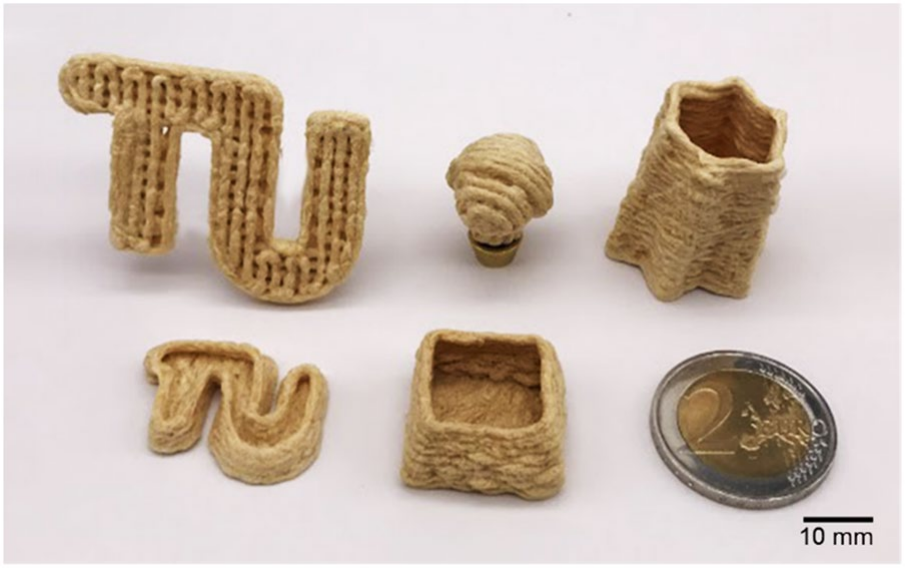
Optical images of the objects printed with F71A29 paste and dried in a freeze dryer (printing conditions: nozzle diameter 1.6 mm, layer height 1.3 mm, printing speed 10 mm/s). The mushroom-shaped object (in the middle in the top) was printed in two parts and then glued together with the same paste and dried again. The size of the objects is compared to a 2 € coin

Compared to other similar printing materials (e.g. clay [26]), the viscosity of fungal pastes was at the lower end of the printable range. This means that pastes flow more easily when there is little pressure in the syringe. In addition to controlling the extrusion pressure precisely, the extruded paste can also be deposited in time by accelerating the movement of the print head. Starting with a 1.6 mm nozzle, a more suitable print speed is 10 mm/s when setting the print height to 1.3 mm. As the used setup is based on continuous extrusion, a fast non-print movement speed could prevent the printed structure from being pulled, and the recommended speed of > 200 mm/s allowed the filament to be torn. 3D parts with different geometries (Fig. 4) were printed with an in-house adapted extrusion-based 3D printer using F71A29 paste. We successfully printed complex shapes with solid squares, hollow vase shapes, and partially filled interiors, etc. Final print heights of up to 39 mm were possible. The tops of printed samples appeared to have slightly shrunk compared to their lower sides. This dimensional difference was caused by the slight deformation on lower layers because of the squeezing from the upper layer, resulting in a larger than set gap between the top layer and the print head, which can be solved by optimizing the printing program.

**Fig. 5:**
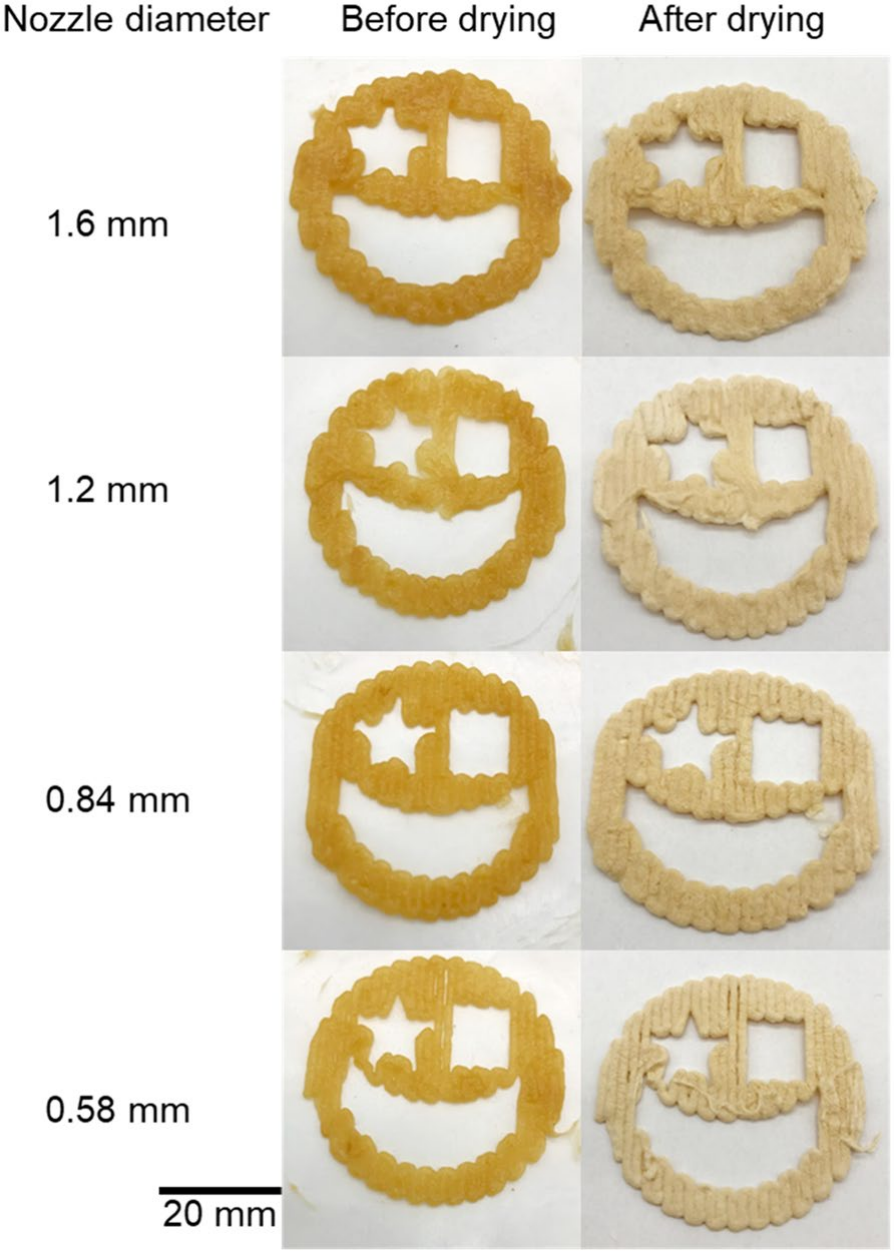
Optical images of objects printed with F71A29 paste using nozzles with diameters from 0.58 to 1.6 mm, layer height from 0.5 to 1.3 mm

The above shapes were obtained by printing with 1.6 mm nozzles. By replacing the nozzle with a finer one, the finest prints could be achieved with a 0.58 mm nozzle (Fig. 5). Compared to the 1.6 mm nozzle results, the 0.58 mm nozzle produced star shapes with more pronounced and sharper corners and a thinner monolayer thickness, which will be suitable for more delicate work.

**Fig. 6:**
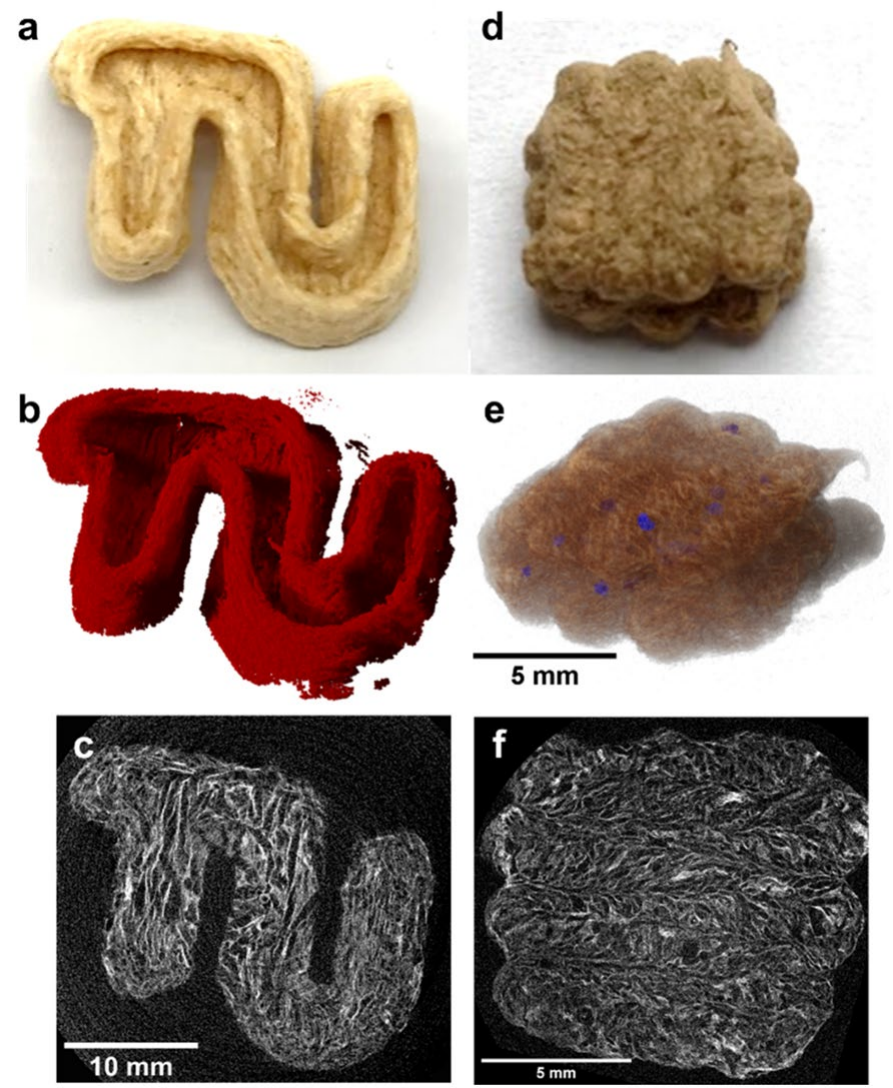
**a** Photographic image of a printed "TU" logo, **b** 3D image of the reconstructed volume, **c** µ-CT tomographic image of a slice through its bottom layer, **d** photographic image of the printed bulk sample, **e** 3D oblique view of the printed bulk sample with enclosed air bubbles in blue and **f** µ-CT tomographic image of a slice through the middle layer of a bulk sample acquired with higher resolution

Using µ-CT measurements (see "Methods"), the inner structure of the printed parts was further investigated. The tomographic section through the printed bottom layer of the "TU" logo revealed a typical freeze-dried or freeze-casted structure with porosity caused by the sublimated ice crystals (Fig. 6a–c) [27, 28]. The attachment of the filaments was investigated for a printed part consisting of three layers of four lanes each. As seen in Fig. 6d–f, the individual lanes were well connected, but can still be easily distinguished by the orientation of the fibers, which remains after freeze-drying, caused by the printing direction. In accordance with SEM results, individual spherical cavities can be detected both within and between the lanes (Fig. 6 f) and are due to enclosed air bubbles.

**Fig. 7:**
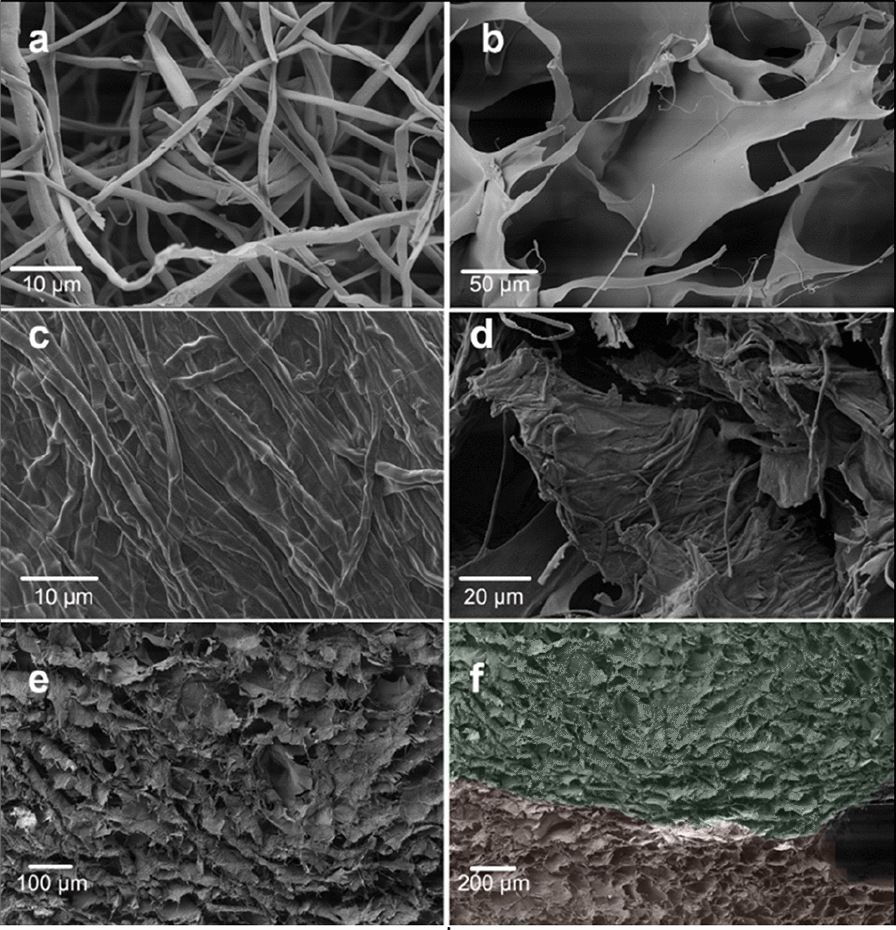
SEM images of **a** cross-section of dried pure mycelium of *F. fomentarius*, **b** cross-section of dried pure alginate, **c** surface view of printed F71A29 (part see Fig. 6d), **d** cross-section of printed F71A29 after CaCl_2_ treatment, **e** foam structure of printed F71A29 and **f** junction between different printed layers (colors are added to differentiate layers)

Microstructural characterization (Fig. 7a) revealed that the *F. fomentarius* mycelium network consists of loose and randomly packed hyphae with diameters of ca. 1–3 µm, while the freeze-dried alginate without mycelium was composed of overlapped microsheets (Fig. 7b). As shown in Fig. 7c, after treatment of the printed F71A29 sample in calcium chloride solution, the mycelium and sodium alginate were tightly bound to form a closed surface. The mycelium fibers and alginate microsheets were well combined to form a mycelium-reinforced composite in the printed F71A29 sample (Fig. 7d). The final printed F71A29 sample resembled a foam structure (Fig. 7e and f) that was formed due to the sublimation of ice crystals during the freeze-drying process. The layers were well attached (Fig. 7f), minuscule air bubbles in the paste were preserved during freeze-drying, forming uniform and smooth cavities inside.

By calculating the ratio of mass and volume, the average bulk densities of dried F71A29 samples (10 mm × 4 mm × 10 mm) were determined to be 0.12 ± 0.01 g/cm^3^. The Young's modulus under compression was 22.21 ± 3.38 kPa and compressive strength (at 10% strain) was 0.51 ± 0.12 MPa, which is comparable to that of expanded polystyrene (density 0.00310–3.50 g/cm^3^, Young’s modulus 0.00650–2.65 GPa, and compressive strength 0.04–10.90 MPa) [29, 30].

### Crosslinking and stability

**Fig. 8:**
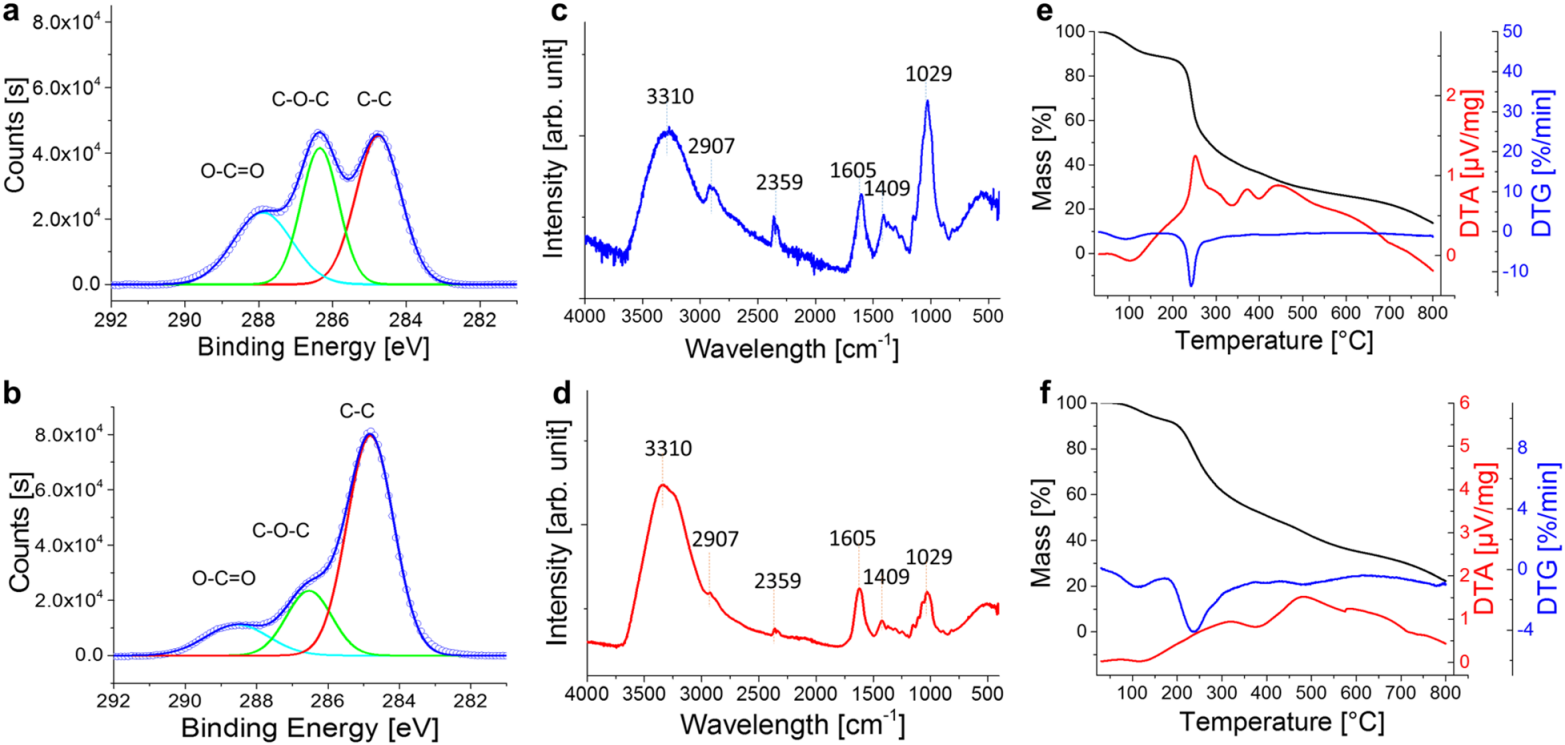
High resolution X-ray photoemission C 1s spectra of F71A29 **a** before and **b** after CaCl_2_ treatment. ATR-FTIR spectra of F71A29 **c** before and **d** after CaCl_2_ treatment. TG, DTG and DTA curves of F71A29 **e** before and **f** after CaCl_2_ treatment carried out in air atmosphere with heating speed 10 °C/min

We further studied the additional crosslinking of the printed parts by adding CaCl_2_ solution to increase the stability of the printed parts. The crosslinking was studied by ATR-FTIR and XPS (see "Methods"), while water immersion tests and TG–DTA (see "Methods") were performed to measure water and thermal stability. As shown in Fig. 8a and b, the plotted C 1s XPS data can be deconvoluted into three peaks corresponding to C–C (284.8 eV), C–O–C or C–OH (286.5 eV), and O–C=O (288.5 eV) [31]. The intensities of C–O–C/C–OH and O–C=O peaks are significantly decreased compared to that of C–C peak after CaCl_2_ treatment, suggesting the successful cross-linkage of alginate molecules with Ca^2+^ ions through the carboxyl and hydroxyl groups [32, 33]. Figure 8c and d show the most relevant ATR-FTIR spectral bands of the printed mycelium-alginate composite before and after CaCl_2_ treatment. Both printed composites showed the characteristic absorption bands that can be attributed to the vibration of O–H (3310 cm^−1^), C–H (2907 cm^−1^), C=C and C≡C (2359 cm^−1^), C=O, C–N, and N–H (1605 cm^−1^), C=O and C–OH (1409 cm^−1^), OC–OH, P=O, and C–C (1029 cm^−1^) [32–35]. The water absorption bands at 3310 cm^−1^ dominated the spectra at higher wavenumbers [36]. The band around 2907 cm^−1^ was mainly because of lipids absorbance [37]. The binding effect of Ca^2+^ on lipids results in the reduction of this band [38]. The peak around 1029 cm^−1^ arises mainly from the vibrations of carbohydrate, and nucleic acid [37]. The spectrum of the sample after CaCl_2_ treatment showed significantly weaker peaks in the region 900–1700 cm^−1^, which is consistent with the XPS results, indicating the reduction of O–C=O groups and confirming the successful cross-linkage of alginate molecules with Ca^2+^ ions.

The crosslinking leads to improved stability of the printed parts in water. While printed parts without CaCl_2_ treatment fell apart after being immersed in water overnight, CaCl_2_-treated printed parts were found to be stable after overnight treatment with water. The influence of the crosslinking step on the thermal stability of printed composites was further studied by thermal gravimetric analysis. Figure 8e and f show TG, DTA, and DTG curves of F71A29 before and after treatment in CaCl_2_ solution. As shown in Fig. 8e, the TG curve of un-crosslinked sample shows a mass loss of 12 wt.% between 30 °C and ~ 170 °C and a corresponding endothermic effect in the DTA data (peak at ~ 101 °C), which presents the loss of water. Other subsequent exothermic peaks between ~ 170 °C and 800 °C indicate the complex multi-decomposition process within fungal-alginate composite at ~ 253 °C, ~ 373 °C, and ~ 444 °C, with a total mass loss of about 86%, which is related to the degradation of organic components, such as polysaccharides and proteins, as well as the degradation of primary residual carbon [39, 40]. For comparison, the thermal analysis of the CaCl_2_ treated sample is shown in Fig. 8f. The TG curve shows a mass loss of 8 wt.% between 30 °C and ~ 180 °C and a corresponding endothermic peak at ~ 115 °C in the DTA curve indicating the loss of water. Subsequent exothermic peaks between ~ 180 °C and 800 °C indicated a complex multi-stage decomposition process within fungal-calcium-alginate composite at ~ 320 °C, ~ 482 °C, and ~ 583 °C due to the combustion of the high organic content. Compared to the untreated printed sample, the decomposition of the cross-linked sample was still a multi-stage process, but with a smoother total mass loss of 78 wt.% till 800 °C, suggesting that crosslinking with calcium can provide higher heat stability to the composite material.

It is worth noting that when CaCl_2_ solution was used on a wet sample directly, the borders of the resulting final product became blurred. However, by firstly freeze-drying un-crosslinked sample, then treating it with CaCl_2_ solution, and freeze-drying it again afterward, the shape of the printed sample was preserved to the fullest extent possible.

## Conclusions

This study reports the successful development of completely bio-based pastes for extrusion-based AM by using fungal mycelium and alginate, which is a common naturally derived rheology agent, as substrates. The pastes of hyphae from *F. fomentarius* combined with alginate and water displayed shear thinning properties which are a prerequisite for 3D printing. They could be extruded through fine nozzle diameters down to 0.58 mm, stacked up to 39 mm, and kept filaments intact with a span of up to 10 mm. The 3D objects obtained displayed complex geometries and maintained their shape after freeze-drying and calcium crosslinking. The properties of the printed objects (i.e. low bulk density of 0.12 ± 0.01 g/cm^3^ and compressive strength) are comparable to those of expanded polystyrene with the additional advantage of being fully biodegradable, which is not the case for expanded polystyrene. In comparison to classical molding techniques, AM facilitates greater flexibility in custom design and allows processing on-demand and locally, making the process an excellent production technology for manufacturing fungal-based materials including, but not limited to, packaging and textiles. As next steps, we will adapt the process to living mycelia of *F. fomentarius* to obtain reactive fungal-based materials which could also be self-healing as well as to develop *F. fomentarius* composite materials to tune their physico-chemical properties and thus open the space for further applications of fungal-based materials.

## Methods

### Chemicals and materials

The following chemicals were used as received without further purification: anhydrous calcium chloride (purity ≥ 97%, Merck), sodium alginate (C_6_H_9_NaO_7_, high viscosity powder with the viscosity of 1319 mPa in 1% aq solution, Alfa Aesar), calcium sulfate dihydrate (purity 98%, Carl Roth, Germany), malt extract agar (Carl Roth, Germany), brown millet (Mühle Schlingemann, Germany) and hemp shives (Hemparade, Netherlands). Deionized water (DIW) was obtained from Merck Millipore.

### *Fomes fomentarius* cultivation

*Fomes fomentarius* strain Papf11 was used for all experiments and obtained from the strain collection of the Chair of Applied and Molecular Microbiology, Technische Universität Berlin. All cultivation procedures have been most recently described in detail (see [18]) and are summarized here in brief: Colonies of *F. fomentarius* were obtained through cultivation on solid complete medium and used to inoculate sterilized millet grains supplemented with 1 wt% calcium sulfate dihydrate. After incubation for 14 days at 25 °C in the dark, the grains were grown through completely and used as “mushroom spawn” to inoculate hemp shives as the solid medium. In doing so, hemp shives were hydrated with 150 wt.% of water in cultivation bags (SacO2, Belgium) and autoclaved. 5 wt.% overgrown millet spawn was added to the wet hemp shives and mixed by kneading. The bags were then heat-sealed and incubated at 25 °C in the dark. After 7 days of incubation the bags were mixed and incubation was continued for another 7 days. The overgrown solid substrate was then crushed using a disinfected shredder (Bosch, Germany) and transferred into a disinfected polypropylene box. After another 19 days of incubation, the culture was removed from the box and dried at 50 °C for three days. Finally, the pure surface mycelium was obtained by careful stripping from the surface.

### Paste preparation

The mycelium was firstly ground with a coffee grinder (Moulinex AR11). Then 500 mg of dried mycelium was dispersed in 10 mL DIW in a 25 mL beaker and mixed with an overhead stirrer (IKA RW16 Basic) with a swiveling blade (TPP Cell Scraper 24 cm). After proper mixing at rotation 1200 min^−1^, alginate was added in different amounts and stirred further to prepare the final paste. The abbreviations for the composition indicate the mass fraction (%) of *F. fomentarius* (F) and alginate (A) within the solid. For example, F71A29 denotes a specimen made from 500 mg *F. fomentarius* and 200 mg alginate, corresponding to 71 wt.% of *F. fomentarius* and 29 wt.% of alginate. The mycelium content was kept at 50 mg/mL in all specimens (Table 1).

**Table 1:** Sample compositions along with the characterization methods applied

Samples	Compositions			Characterization				
	Mass (*F. fomentarius*) mg	Mass (Alginate) mg	Volume(H_2_O) mL	Rheology	Homogeneity	Collapse	Stackability	Microstructures
F91A09	50	5	10	×	×			
F83A17	50	10	10	×	×	×		
F77A23	50	15	10	×	×			
F71A29	50	20	10	×	×	×	×	×
F62A38	50	30	10	×	×	×		
F56A44	50	40	10	×	×	×		
F00A09	0	5	10	×				
F00A17	0	10	10	×		×		
F00A23	0	15	10	×				
F00A29	0	20	10	×		×		
F00A38	0	30	10	×		×		
F00A44	0	40	10	×		×		

### Rheological tests

To be suitable for the extrusion-based AM, the pastes should possess a high initial viscosity with shear-thinning behavior, in an ideal case recovering its initial viscosity after extrusion. Two rheological tests were conducted at room temperature to explore the rheological properties of the developed pastes: (i) steady-state flow tests and (ii) three-step flow tests under a certain (fixed) shear rate simulating the extrusion process for AM [25]. For steady-state flow tests, the viscosity over the shear rate (0.1 to 100 s^−1^) was measured with an Anton Paar MCR 301 rheometer and a plate-plate measurement system D-CP/PP25 (Ø 25 mm and 0.5 mm gap). For the three-step flow test, at step I, to mimic the shear conditions before extrusion, the pastes were then subjected to a low shear rate of 0.1 s^−1^ for 60 s to simulate at-rest state prior to extrusion, (since it was not possible to measure the viscosity at true zero-shear conditions due to the rheometer specifications). At step II, the shear rate was increased to 100 s^−1^ and held for 10 s. This simulates the condition of the paste under a specific shear rate during the printing process. And finally, at step III, the shear rate was lowered to 0.1 s^−1^ and held for 60 s to simulate the final state after the extrusion. The viscosity recovery (%) is described as the ratio of the value of viscosity in step III to its initial value in step I.

### Extrusion (3D printing)

#### Printer settings

**Fig. 9:**
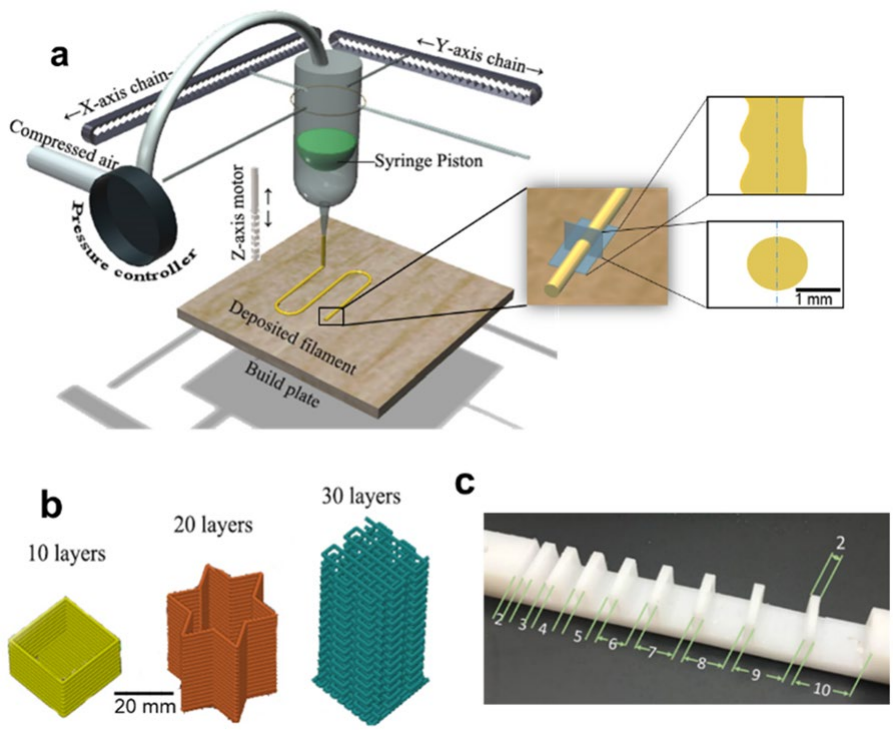
**a** Schematic diagram of the in-house adapted 3D printer for extrusion-based AM and the morphology of the filaments after being extruded and deposited on the substrate. **b** Schematic diagram of AM models for stackability test and **c** the dimensions (in mm) of the filament collapse test pillar plate

An in-house adapted Fused Filament Fabrication printer Ultimaker 2 Go (Netherlands) was used. The printing head was replaced with a syringe holder, also obtained by 3D printing, and the syringe was then connected to an independent gas supply system (Fig. 9a). When the print head runs on the set trajectory, speed (4–10 mm/s), and layer height, the printer’s compressed air valve is opened, and the piston inside the syringe pushes the paste out of the nozzle (diameter 0.58–1.6 mm). The extrusion rate was able to be controlled by tuning the pressure inside the syringe.

#### Homogeneity of extruded filaments

Aiming at obtaining consistent structure with strong adhesion between lanes and layers, the random variations in the radial direction of the extruded filaments *(*Fig. 9a) were studied in dependence of the paste composition (from 91 to 56 wt.% mycelium). Thus, filament widths were measured at 20 different points per filament per paste, after extruding the pastes through a 1.2 mm nozzle diameter syringe by hand. Width variance from the nozzle diameters was then ascertained to evaluate the irregularity of the filament.

#### Stackability and filament collapse tests

To test the stackability of pastes, objects with different shapes/geometry were fabricated with a printing speed of 10 mm/s, nozzle diameter of 1.6 mm, and a layer height of 1.3 mm. Constructs in the shape of squares, stars, and grids were stacked with 10, 20, and 30 layers, respectively (Fig. 9b). This process was carried out at least three times for each shape. Filament collapse tests were conducted to determine the shape fidelity and the effect of pore size on printability when the interior was not 100% filled [41]. A pillar plate with gap distances from 2 to 10 mm was built (Fig. 9c). Pastes of varying mycelium content ranging from 83 to 56 wt.% were extruded under compressed air using a syringe with a nozzle diameter of 1.2 mm moved by hand. A single filament was deposited on the pillars for each composition, and the formed filaments were then evaluated from the side.

#### Fabrication and post-processing

After printing with the settings as mentioned in 2.5.1, the samples were freeze-dried in a Modulyo 4 K freeze dryer (Edwards, UK) at − 50 °C, 0.05 mbar for 24 h. To increase the stability of the printed parts, they were dipped in 4 wt.% CaCl_2_ solutions for cross-linking the alginate component afterward and freeze-dried again under the same conditions described above.

### Characterization

The microstructures of printed samples were analyzed by scanning electron microscopy (Zeiss Leo Gemini 1530) with a secondary electron detector after coating them with gold. The electron high tension voltage, aperture size, and working distance used in the imaging were 3.00 kV/5.00 kV, 30.00 mm, and 7.4 mm/9.4 mm, respectively, and magnifications were ×100, ×200, ×500, and ×5000 scale. X-ray tomographic measurements (µ-CT) were performed on a self-developed setup consisting of an L8121-03 microfocus X-ray tube and a C7942CA-02 flat panel detector (both Hamamatsu, Japan) with an accelerating voltage of 80 kV [42]. The magnification was adjusted to a resulting pixel size of 16.5 µm and the volume was reconstructed using a filtered back-projection cone-beam algorithm with Octopus 8.8 (Inside Matters, Belgium).

The compressive strength of six printed samples using F71A29 paste was measured based on the ISO 604/ASTM D695. The samples (10 mm × 10 mm × 4 mm) were printed with a nozzle diameter of 1.6 mm, layer height 1.3 mm, printing speed 10 mm/s, filled, and then sanded to obtain parallel surfaces. As fungal materials do not rupture, their compressive strength can be determined at a 10% deformation [43]. Tests were performed using a universal testing machine Zwick/Roell Z020 with a testing speed of 1.27 mm min^−1^. Young’s modulus was determined from the slope of a uniaxial stress–strain curve. The bulk density of the object was obtained by dividing its mass by its volume.

Fourier transform infrared spectroscopy (FTIR) in Attenuated Total Reflection (ATR) mode was carried out in a Vertex 70 (Bruker, Germany) in the range of 400–4000 cm^−1^ for identification of distinct functional groups. X-ray photoelectron spectroscopy (XPS) measurements were performed on a K-alpha (Thermo Fischer Scientific, USA) equipped with a monochromatic Al Kα source. Carbon pads were used to hold the samples. Scans were taken in the constant analyzer energy mode with a step size of 0.1 eV, pass energy of 50 eV, and spot size of 400 µm. The C 1s core line with a binding energy of 284.8 eV was used to calibrate all XPS spectra.

To study the thermal stability and decomposition of the printed objects before and after CaCl_2_ treatment, thermal gravimetry analysis (TGA) and differential thermal analysis (DTA) were carried out under synthetic air flow using STA 449F3 (Netzsch, Germany) up to 800 °C with heating rate of 10 °C/min. The samples were stored in a desiccator before thermal analysis.

## Supplementary Information

**Additional file 1: Fig. S1.** Rheological properties of pure alginate gel pastes: a) results of the flow tests and b) results of the three-step flow recovery tests.

## Data Availability

The datasets during and/or analysed during the current study available from the corresponding author on reasonable request.

## Abbreviations

AM: Additive manufacturing
ATR: Attenuated Total Reflection
DIW: Deionized water
DTA: Differential thermal analysis
DTG: Derivative thermogravimetry
*F. fomentarius*: *Fomes fomentarius*
FTIR: Fourier transform infrared spectroscopy
µ-CT: Micro-computed tomography
SEM: Scanning electron microscopy
TG: Thermogravimetric analysis
XPS: X-ray photoelectron spectroscopy

## References

[1] Islam MR, Tudryn G, Bucinell R, Schadler L, Picu RC (2018). Stochastic continuum model for mycelium-based bio-foam. Mater Des.

[2] Meyer V, Basenko EY, Benz JP, Braus GH, Caddick MX, Csukai M, de Vries RP, Endy D, Frisvad JC, Gunde-Cimerman N (2020). Growing a circular economy with fungal biotechnology: a white paper. Fungal Biol Biotechnol.

[3] Joshi K, Meher MK, Poluri KM (2020). Fabrication and characterization of bioblocks from agricultural waste using fungal mycelium for renewable and sustainable applications. ACS Appl Bio Mater.

[4] Jones M, Mautner A, Luenco S, Bismarck A, John S (2020). Engineered mycelium composite construction materials from fungal biorefineries: a critical review. Mater Des.

[5] Pelletier MG, Holt GA, Wanjura JD, Bayer E, McIntyre G (2013). An Evaluation study of mycelium based acoustic absorbers grown on agricultural by-product substrates. Ind Crops Prod.

[6] Mosler J Fungus as a sound absorber. 3 Research News, Fraunhofer, January 4, 2021, https://www.fraunhofer.de/en/press/research-news/2021/january-2021/fungus-as-a-sound-absorber.html

[7] Sekar V, Fouladi MH, Namasivayam SN, Sivanesan S (2019). Additive manufacturing: a novel method for developing an acoustic panel made of natural fiber-reinforced composites with enhanced mechanical and acoustical properties. J Eng.

[8] Jones M, Bhat T, Kandare E, Thomas A, Joseph P, Dekiwadia C, Yuen R, John S, Ma J, Wang C-H (2018). Thermal degradation and fire properties of fungal mycelium and mycelium—biomass composite materials. Sci Rep.

[9] Sathiyaseelan A, Shajahan A, Kalaichelvan PT, Kaviyarasan V (2017). Fungal chitosan based nanocomposites sponges—an alternative medicine for wound dressing. Int J Biol Macromol.

[10] Henning LM, Simon U, Abdullayev A, Schmidt B, Pohl C, Guitar TN, Vakifahmetoglu C, Meyer V, Bekheet MF, Gurlo A (2021). Effect of fomes fomentarius cultivation conditions on its adsorption performance for anionic and cationic dyes. ACS Omega.

[11] Jones M, Gandia A, John S, Bismarck A (2021). Leather-like material biofabrication using fungi. Nat Sustain.

[12] Cerimi K, Akkaya KC, Pohl C, Schmidt B, Neubauer P (2019). Fungi as source for new bio-based materials: a patent review. Fungal Biol Biotechnol.

[13] Manan S, Ullah MW, Ul-Islam M, Atta OM, Yang G (2021). Synthesis and applications of fungal mycelium-based advanced functional materials. J Bioresour Bioprod.

[14] Tišma M, Žnidaršič-Plazl P, Šelo G, Tolj I, Šperanda M, Bucić-Kojić A, Planinić M (2021). Trametes versicolor in lignocellulose-based bioeconomy: state of the art challenges and opportunities. Bioresour Technol.

[15] Ross, P.; Francisco, S.; Scullin, M.; Francisco, S.; Wenner, N.; Chase, J.; Miller, Q.; Saltidos, R.; Francisco, S.; McGaughy, P.Mycelium growth bed with perforation layer and related method for creating a uniform sheet of mycelium from a solid-state medium. https://patents.google.com/patent/US20200196541A1/en

[16] Raudaskoski M (2019). The central role of septa in the basidiomycete schizophyllum commune hyphal morphogenesis. Fungal Biol.

[17] Synytsya A, Novák M (2013). Structural diversity of fungal glucans. Carbohyd Polym.

[18] Mind the Fungi (2020), Edited by Vera Meyer and Regine Rapp. Universitätsverlag TU Berlin. ISBN 978-3-7983-3168-6

[19] Bhardwaj A, Vasselli J, Lucht M, Pei Z, Shaw B, Grasley Z, Wei X, Zou N (2020). 3D Printing of biomass-fungi composite material: a preliminary study. Manuf Lett.

[20] Blast Studio - 3d Printed Mycelium Objects. https://www.blast-studio.com. Accessed 6 July 2021.

[21] Burry J, Sabin JE, Sheil B, Skavara M (2020) Fabricate 2020. UCL Press https://discovery.ucl.ac.uk/id/eprint/10094460/1/Fabricate-2020.pdf

[22] Tavares, F. Could Future Homes on the Moon and Mars Be Made of Fungi? http://www.nasa.gov/feature/ames/myco-architecture. Accessed 8 July 2021.

[23] Wösten HAB, Krijgsheld P, Montalti M, Läkk H Growing Fungi Structures in Space. 17 https://www.esa.int/gsp/ACT/doc/ARI/ARI%20Study%20Report/ACT-RPT-HAB-ARI-16-6101-Fungi_structures.pdf

[24] Balla VK, Kate KH, Satyavolu J, Singh P, Tadimeti JGD (2019). Additive manufacturing of natural fiber reinforced polymer composites: processing and prospects. Compos B Eng.

[25] Li H Study of hydrogels for 3D printing of constructs with strong interfacial bonding. 2018, https://hdl.handle.net/10356/82592 Nanyang Technological University

[26] Feilden, E. Additive manufacturing of ceramics and ceramic composites via robocasting. 200.

[27] Bahrami A, Simon U, Soltani N, Zavareh S, Schmidt J, Pech-Canul MI, Gurlo A (2017). Eco-fabrication of hierarchical porous silica monoliths by ice-templating of rice husk ash. Green Chem.

[28] Shao G, Hanaor DAH, Shen X, Gurlo A (2020). Freeze casting: from low-dimensional building blocks to aligned porous structures—a review of novel materials, methods, and applications. Adv Mater.

[29] Negussey D, Jahanandish M. Comparison of some engineering properties of expanded polystyrene with those of soils (with discussion and closure). Transportation Research Record, 1993 (1418).

[30] Overview of Materials for Expanded Polystyrene (EPS): http://www.matweb.com/search/DataSheet.aspx?MatGUID=5f099f2b5eeb41cba804ca0bc64fa62f&ckck=1 Accessed 13 Sep 2021.

[31] Shao G, Hanaor DAH, Wang J, Kober D, Li S, Wang X, Shen X, Bekheet MF, Gurlo A (2020). Polymer-derived SiOC integrated with a graphene aerogel as a highly stable Li-Ion battery anode. ACS Appl Mater Interfaces.

[32] Song D, Park S-J, Kang HW, Park SB, Han J-I (2013). Recovery of Lithium(I), Strontium(II), and Lanthanum(III) Using Ca–Alginate Beads. J Chem Eng Data.

[33] Kühbeck D, Mayr J, Häring M, Häring M, Quignard F, Díaz DD (2015). Evaluation of the nitroaldol reaction in the presence of metal ion-crosslinked alginates. New J Chem.

[34] Torres E, Mata YN, Blázquez ML, Muñoz JA, González F, Ballester A (2005). Gold and silver uptake and nanoprecipitation on calcium alginate beads. Langmuir.

[35] Lecellier A, Mounier J, Gaydou V, Castrec L, Barbier G, Ablain W, Manfait M, Toubas D, Sockalingum GD (2014). Differentiation and identification of filamentous fungi by high-throughput FTIR spectroscopic analysis of mycelia. Int J Food Microbiol.

[36] Salman A, Tsror L, Pomerantz A, Moreh R, Mordechai S, Huleihel M (2010). FTIR spectroscopy for detection and identification of fungal phytopathogenes. Spectroscopy.

[37] Sivakesava S, Irudayaraj J, DebRoy C (2004). Differentiation of microorganisms by FTIR-ATR and NIR spectroscopy. Trans ASAE.

[38] Feigenson GW (1989). Calcium ion binding between lipid bilayers: the four-component system of phosphatidylserine, phosphatidylcholine, calcium chloride, and water. Biochemistry.

[39] Jones M, Weiland K, Kujundzic M, Theiner J, Kählig H, Kontturi E, John S, Bismarck A, Mautner A (2019). Waste-derived low-cost mycelium nanopapers with tunable mechanical and surface properties. Biomacromol.

[40] Soares JP, Santos JE, Chierice GO, Cavalheiro ETG (2004). Thermal behavior of alginic acid and its sodium salt. Eclet Quím.

[41] Therriault D, White SR, Lewis JA (2007). Rheological behavior of fugitive organic inks for direct-write assembly. Appl Rheol.

[42] García Moreno F, Fromme M, Banhart J (2004). Real-time X-ray radioscopy on metallic foams using a compact micro-focus source. Adv Eng Mater.

[43] Vakifahmetoglu C, Semerci T, Soraru GD (2020). Closed porosity ceramics and glasses. J Am Ceram Soc.

