# Supplementary material for: Extrusion-based additive manufacturing of fungal-based composite materials using the tinder fungus Fomes fomentarius
Source: Fungal Biol Biotechnol. 2021 Dec 21;8:21. doi: 10.1186/s40694-021-00129-0 (PMC8693477; doi:10.1186/s40694-021-00129-0)
Supplement: Supplementary file 1 — Additional file 1: Fig. S1. Rheological properties of pure alginate gel pastes: a) results of the flow tests and b) results of the three-step flow recovery tests. [file 40694_2021_129_MOESM1_ESM.docx]

***Supplementary materials***

**Extrusion-based additive manufacturing of fungal-based composite materials using the tinder fungus *Fomes fomentarius***

Huaiyou Chen^a^, Amanmyrat Abdullayev^a^, Maged F. Bekheet^a^, Bertram Schmidt^b^, Isabel Regler^b^, Carsten Pohl^b^, Cekdar Vakifahmetoglu^c^, Mathias Czasny^a^, Paul H. Kamm^d^, Vera Meyer^b^, Aleksander Gurlo^a^, Ulla Simon^a,^*

^a^ Technische Universität Berlin, Faculty III Process Sciences, Institute of Material Science and Technology, Chair of Advanced Ceramic Materials, Straße des 17. Juni 135, 10623 Berlin, Germany

^b^ Technische Universität Berlin, Faculty III Process Sciences, Institute of Biotechnology, Chair of Applied and Molecular Microbiology, Straße des 17. Juni 135, 10623 Berlin, Germany

^c^ Department of Materials Science and Engineering, Izmir Institute of Technology, 35430, Urla, Izmir, Turkey

^d^ Helmholtz-Zentrum Berlin für Materialien und Energie, Institute of Applied Materials, Hahn-Meitner-Platz 1, 14109 Berlin, Germany

* Corresponding author. Contact details: ulla.simon@ceramics.tu-berlin.de


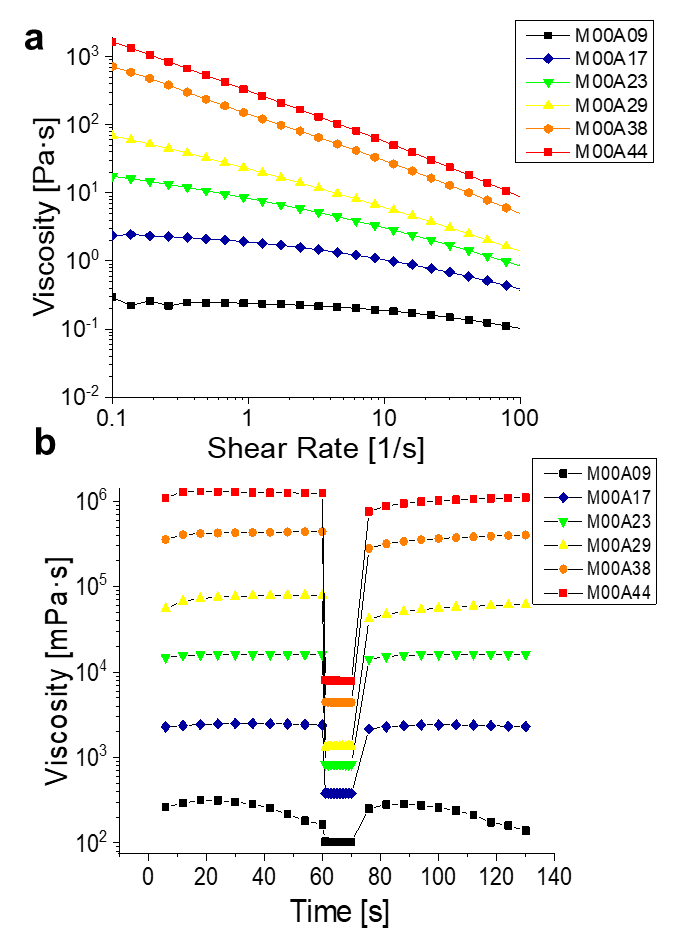


*Figure S1 Rheological properties of pure alginate gel pastes: a) results of the flow tests and b) results of the three-step flow recovery tests.*
